# Functional characterization of AsOBP21f, an odorant-binding protein involved in human odor detection in the malaria vector *Anopheles sinensis*

**DOI:** 10.1186/s13071-026-07333-0

**Published:** 2026-03-23

**Authors:** Fang Li, Ou Xu, Jiajia Zhou, Shulin He, Bin Chen, Zhengbo He

**Affiliations:** 1 Chongqing Key Laboratory of Vector Control and Utilization, Institute of Entomology and Molecular Biology, Chongqing Normal University, Chongqing, 401331 China; 2 Chongqing Collaborative Innovation Center for Child Nutrition and Health Development, Chongqing University of Education, Chongqing, 400067 China

**Keywords:** *Anopheles sinensis*, *AsOBP21f*, Human odor, Host seeking, RNA interference

## Abstract

**Background:**

Olfactory detection of host odors is fundamental to mosquito host-seeking behavior. Although the olfactory pathways of model species such as *Anopheles gambiae* have been well characterized, the molecular basis of human odor detection in *Anopheles sinensis*, an important malaria vector in Asia with opportunistic feeding habits, remains poorly understood. This study systematically investigates the functional role of the odorant-binding protein AsOBP21f in this process.

**Methods:**

*AsOBP21f* was cloned and characterized using bioinformatics, quantitative reverse transcription PCR (RT-qPCR), and phylogenetic analyses. Recombinant AsOBP21f protein was expressed and purified for fluorescence competitive binding assays with 35 human odorants. Molecular docking was performed to elucidate ligand-binding interactions. Electroantennogram (EAG) recordings and behavioral assays were conducted to evaluate mosquito responses to high-affinity ligands. RNA interference (RNAi) knockdown was used to assess the functional role of *AsOBP21f* in host-seeking and blood-feeding behavior.

**Results:**

*AsOBP21f* was predominantly expressed in olfactory tissues of mosquito females, including the antennae and proboscis. Its protein exhibited notable selectivity for hydrophobic odor molecules with C10–C15 carbon chains, and had strong binding affinities for methyl tridecanoate, dodecanal, decanal, and pentadecanoic acid. Behavioral experiments further demonstrated dose-dependent effects of these ligands; methyl tridecanoate showed significant attraction, while dodecanal exhibited clear repellency. RNAi-mediated silencing of *AsOBP21f* significantly reduced the antennal electrophysiological response of mosquito females to host odors and markedly decreased blood-feeding success.

**Conclusions:**

These results highlight the role of *AsOBP21f* in host-seeking behavior through the detection of human odors, such as methyl tridecanoate, in *An. sinensis*, providing a potential target for malaria control.

**Graphical Abstract:**

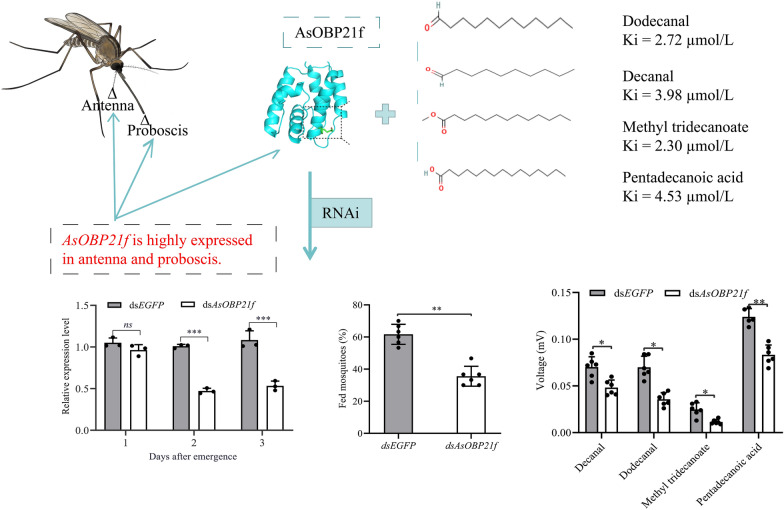

**Supplementary Information:**

The online version contains supplementary material available at 10.1186/s13071-026-07333-0.

## Background

Blood-feeding on vertebrate hosts is crucial for mosquito reproduction and simultaneously facilitates pathogen transmission. The host-seeking process, which initiates mosquito blood-feeding behavior, relies on the integration of multiple sensory cues including olfactory, visual, thermal, and humidity signals [1–4]. Among these, olfaction plays the predominant role in detecting host-emitted volatile compounds [5]. Human body odor contains various components, such as carboxylic acids (e.g., lactic acid, octanoic acid), alcohols (e.g., 1-octen-3-ol, 1-tetradecanol, 3-methyl-1-butanol, octanol), aldehydes (e.g., heptanal), and phenolic or aromatic compounds (e.g., indole), all of which can elicit either attractive or repellent responses in mosquitoes [6–8]. Some of these compounds, including 1-octen-3-ol, lactic acid, and octanol, have already been utilized as attractants in mosquito surveillance and control programs [9, 10]. Furthermore, mosquito host preferences are influenced by the specific composition and concentration of odorants emitted by different hosts, with significant variation observed among species [11–13]. For example, *An. gambiae* demonstrates strong anthropophilic tendencies, while *An. sinensis* exhibits more opportunistic feeding behavior, targeting both humans and livestock. Therefore, a deeper understanding of the interactions between human odorants and the mosquito olfactory system is crucial for elucidating the molecular mechanisms underlying mosquito host-seeking behavior.

The primary olfactory organs of mosquitoes include the antennae, maxillary palps, and proboscis, which bear various sensilla capable of responding to diverse external stimuli [14–17]. Within these sensilla, an array of olfactory-related proteins, including but not limited to chemosensory proteins (CSPs), odorant-binding proteins (OBPs), odorant receptors (ORs), gustatory receptors (GRs), ionotropic receptors (IRs), sensory neuron membrane proteins (SNMPs), and odorant-degrading enzymes (ODEs), facilitate odor detection [18]. OBPs, a class of small, water-soluble acidic proteins, recognize and transport hydrophobic odorant molecules across the aqueous lymph to specific ORs on the sensory neuron membrane, thereby initiating the olfactory signal transduction cascade [19].

Mosquito OBPs play a crucial role in host-seeking behavior by recognizing specific host-derived odorants. For instance, *An. gambiae* AgamOBP1 binds to indole, a key component of human odor. RNAi-mediated knockdown of AgamOBP1 significantly reduced the electroantennogram (EAG) response to indole, suggesting its functional role in olfactory detection [20]. Similarly, *Aedes aegypti* AaegOBP22 showed predominant expression in antennae and salivary glands, and its suppression by RNAi resulted in markedly impaired blood-feeding behavior [21]. In addition, both AalbOBP37 and AalbOBP39 can bind to indole and trimethylindole in *Aedes albopictus*; the knockdown of both genes not only attenuated EAG responses to these compounds but also significantly altered attraction/repellent behaviors [22]. AsOBP1, an antennae-specific protein in *An. sinensis*, strongly bound heptanal and 1-tetradecanol. Its knockout disrupted host-seeking and blood-feeding in females, while topical application of these ligands boosted mosquito attraction to mice [8].

*Anopheles sinensis* (Diptera: Culicidae) is a major vector of *Plasmodium vivax* in East and Southeast Asia. Previous genomic analyses identified 64 odorant-binding protein (OBP) genes in *An. sinensis*, which were classified into three subfamilies: Classic, Plus-C, and Atypical [23]. Among these genes, *AsOBP21f* was found to be highly expressed in the antennae and proboscis of female mosquitoes [24]. Given this female-biased expression pattern in key olfactory organs, we hypothesized that AsOBP21f may play a role in host-seeking and other olfaction-mediated behaviors. To evaluate this hypothesis, we employed multiple experimental approaches to characterize the function of AsOBP21f and its contribution to the olfactory mechanisms underlying host-seeking in *An. sinensis*.

## Methods

### Mosquito strains

The mosquitoes used in this study were kept in the Institute of Entomology and Molecular Biology at Chongqing Normal University. The populations of wild-type strain (Wuxi) of *An. sinensis* and the homozygous knockout mutant strain of the *AsOrco* gene (*AsOrco*^−/−^) generated from the Wuxi strain [25] were reared in a climate-controlled chamber at 27 ± 1 °C, 75 ± 5% relative humidity, and a photoperiod of 12L:12D (light phase: 09:00–21:00). Adults were routinely maintained on a 10% (w/v) glucose solution, and female adults at day 3 after emergence were blood-fed with anesthetized mice for about 20 min. Blood-feeding on mice was approved and monitored by The Animal Care and Use Committee of Chongqing Normal University (protocol ACUC201003).

### Cloning and bioinformatics analysis of AsOBP21f

On the basis of previously identified *AsOBP21f* gene sequence [23], we designed specific primers (Table S1) and verified the *AsOBP21f* gene sequence by sequencing the PCR products amplified from cDNA of *An. sinensis* females as template. The sequencing was carried out at Shanghai Sangon Biotechnology Co., Ltd. The verified sequence of *AsOBP21f* was used for an online BLAST search on VectorBase (https://www.vectorbase.org/blast) to obtain the corresponding genomic sequence. The molecular weight and isoelectric point of the protein were predicted using the ExPASy online tool (https://www.expasy.org/compute_pi/). Protein domains were analyzed using the SMART tool (https://smart.embl-heidelberg.de/); signal peptides were predicted with the SignalP 5.0 Server (https://www.cbs.dtu.dk/services/SignalP/). Homologous sequences of other mosquitoes were retrieved from VectorBase using the nucleotide and protein sequences of *AsOBP21f* as query sequences. A phylogenetic tree was constructed on the basis of amino acid sequences using the neighbor-joining (NJ) method in MEGA 4.0 with 1000 bootstrap replicates.

### Expression analysis of the AsOBP21f gene

To validate the transcriptomic expression data [24], we performed quantitative reverse transcription PCR (RT-qPCR) to quantify *AsOBP21f* expression in the major olfactory tissues of male and female adult *An. sinensis*. Antennae, proboscises, and maxillary palps from 3-day-old adults were collected, and total RNA was extracted using the Ultrapure RNA Kit (CWBIO, Beijing, China). The RNA was then treated with DNase I (Promega, Madison, WI, USA) to remove residual genomic DNA according to the manufacturers’ instructions. First-strand cDNA was synthesized from 1 μg of total RNA using the GoScript™ Reverse Transcription System (Promega, Madison, WI, USA). RT-qPCR was performed on a StepOnePlus™ Real-Time PCR System (Applied Biosystems, Foster City, USA) using the UltraSYBR Mixture (CWBIO, Beijing, China). Each 10 μL reaction contained 5 μL of 2× SYBR Green Mix, 0.2 μL of each gene-specific primer (10 µmol/L), and 4.6 μL of cDNA equivalent to 50 ng of total RNA. The thermal cycling conditions were set as follows: 95 °C for 2 min, followed by 40 cycles of 95 °C for 10 s, 60 °C for 10 s, and 72 °C for 20 s. Three biological replicates were conducted for each tissue, with three technical replicates for each biological replicate. The ribosomal protein S7 (*Rps7*) gene was used as the internal reference; relative expression levels were calculated using the 2^−ΔΔCt^ method. Primer sequences used for RT-qPCR are listed in Table S1.

### Prokaryotic expression and purification of the AsOBP21f

To express the AsOBP21f recombinant protein, gene-specific primers were designed on the basis of the *AsOBP21f* coding sequence using Primer Premier 5.0. Restriction enzyme sites for *Nco*I *and Xho*I were incorporated at the 5′ ends of the forward and reverse primers, respectively. The coding region of *AsOBP21f*, excluding the signal peptide, was amplified by PCR using cDNA synthesized from 3-day-old mosquito females as template. The amplified product and the pET-28a (+) vector were digested with *Nco*I and *Xho*I, purified, and ligated using T4 DNA ligase. The recombinant plasmid was then transformed into competent *Escherichia coli* BL21 (DE3) cells. Positive clones were identified by PCR and further verified by DNA sequencing. The target clone was subsequently inoculated into LB medium supplemented with ampicillin and cultured at 37 °C with shaking at 195 rpm until the OD_600_ reached 0.6. Protein expression was then induced by adding IPTG to a final concentration of 0.5 mmol/L, followed by incubation at 37 °C for 5 h. Cells were harvested by centrifugation, lysed by ultrasonication in the NTA-0 buffer (50 mmol/L Tris–HCl 8.0 + 0.2 mol/L NaCl), and separated into soluble (supernatant) and insoluble (pellet) fractions by centrifugation. The presence of recombinant AsOBP21f in both fractions was determined by SDS–PAGE analysis. The primer sequences used for prokaryotic expression vector construction are listed in Table S1.

Protein purification was performed using nickel–nitrilotriacetic acid (Ni–NTA) affinity chromatography according to the manufacturer’s protocol. Briefly, the sample was loaded on the Ni–NTA column pre-equilibrated with 0.2 M nickel sulfate. The target protein was eluted using 20 mM imidazole solution and then desalted and concentrated using a desalting column (ThermoFisher Scientific, Waltham, MA). The protein concentration was determined using the Micro BCA Protein Assay Kit (Cwbio, Beijing, China). Purified proteins were aliquoted and stored at −80 °C for subsequent use.

### Fluorescence competitive binding assay

A fluorescence competitive binding assay was conducted to evaluate the binding affinity between AsOBP21f and a set of human-derived odorants. Based on human odorants identified by gas chromatography-mass spectrometry (GC/MS) [26] and electrophysiological analyses [2], we selected 35 human-derived compounds that elicited strong electrophysiological responses for use in fluorescent competitive binding assays (Table 1). The assays were performed using a Hitachi F-2500 fluorescence spectrophotometer equipped with a 1-cm pathlength quartz cuvette and a slit width of 10 nm. The protocol was based on our previously described method with minor modifications [8]. The binding constant of AsOBP21f to the fluorescent probe 1-NPN was determined at a 337-nm excitation wavelength with emission spectra recorded from 390 to 500 nm. Initially, 50 mmol/L Tris buffer (pH 7.4) was added to the quartz cuvette, followed by the addition of AsOBP21f protein to a final concentration of 2 μmol/L. After mixing thoroughly and incubating for 2 min at room temperature, we recorded the emission spectrum. Subsequently, 1-NPN was added stepwise to final concentrations ranging from 2 to 16 μmol/L, with 2 μmol/L increments. Each measurement was repeated three times. The binding constant (*K*_1-NPN_) for the AsOBP21f/1-NPN complex was calculated using the Scatchard equation.

**Table 1:** Competitive binding affinity of selected ligands with AsOBP21f

	Odorants	CAS No.	Purity (%)	Molecular weight	IC_50_ (µmol/L)	*K*i (μmol/L)
Carboxylic acids	Lauric acid	143-07-7	98.0	200.31	25.24	17.75
	Undecanoic acid	112-37-8	99.0	186.29	20.46	14.39
	Pentadecanoic acid	1002-84-2	98.0	242.39	6.43	4.53
	Tridecanoic acid	638-53-9	98.0	214.34	14.73	10.36
	n-Hexanoic acid	142-62-1	99.5	116.16	–	–
	Decanoic acid	334-48-5	99.0	172.26	–	–
Ketones	2-Pentanone	107-87-9	99.0	86.13	–	–
	3-Pentanone	96-22-0	98.0	86.13	–	–
	Methyl heptenone	110-93-0	98.0	126.2	–	–
Esters	Methyl tridecanoate	1731-88-0	99.0	228.36	3.26	2.30
	Methyl nonanoate	1731-84-6	99.0	172.26	26.26	18.27
– -	–	120-72-9	99.0	117.15	–	–
	–	83-34-1	98.0	131.17	–	–
	4-(Aminomethyl)piperidine	7144-05-0	98.0	114.10	–	–
	*para*-xylene	106-42-3	99.8	106.17	–	–
	*trans*-2-Octene	13389-42-9	99.5	112.21	–	–
	*trans*-3-Octene	14919-01-8	99.5	112.21	–	–
	n-Propylbenzene	103-65-1	99.7	120.19	15.58	10.96
	Cumarin	91-64-5	99.0	146.14	–	–
	2-Methylpyridine	109-06-8	98.0	93.13	–	–
Aldehydes	Nonanal	124-19-6	96.0	142.24	–	–
	Octanal	124-13-0	99.0	128.21	–	–
	2-Methylbutanal	96-17-3	98.0	86.13	–	–
	Decanal	112-31-2	97.0	156.27	5.65	3.98
	Hexanal	66-25-1	99.0	100.16	–	–
	Butanal	123-72-8	99.5	72.11	–	–
	Heptanal	111-71-7	98.0	114.19	–	–
	Dodecanal	112-54-9	95.0	184.32	3.87	2.72
	Undecanal	112-44-7	98.0	170.29	18.27	12.71
Alcohols	1-Octen-3-ol	3391-86-4	98.0	128.21	–	–
	2-Ethyl-1-hexanol	104-76-7	99.5	130.23	–	–
	*trans*-2-Octen-1-ol	18409-17-1	95.0	128.21	–	–
	*cis*-2-Hexen-1-oL	928-94-9	92.0	100.16	–	–
	2-Decanol	1120-06-5	98.0	158.28	–	–
	1-Tetradecanol	112-72-1	99.5	214.38	–	–

*K*i, dissociation constant. If a ligand had a *K*i value greater than 30 µmol/L, we considered that AsOBP21f did not bind to that ligand; *K*i values not calculated are represented as “–”

To assess the binding affinity of AsOBP21f to selected odorants, each odorant was first dissolved in methanol to a final concentration of 1 mmol/L. The addition of 50 mmol/L Tris Buffer (pH 7.4) to the quartz cuvette was followed the fluorescent probe 1-NPN and the AsOBP21f protein to reach final concentrations of 2 μmol/L each. Each odorant solution was added incrementally to final concentrations of 10, 20, 30, 40, 50, 60, and 70 μmol/L; the maximum fluorescence intensity was recorded after each addition. Each assay was performed in triplicate. The dissociation constant (*K*i) of each odorant–AsOBP21f combination was calculated using the following formula: *K*i = [IC_50_]/(1 + [1-NPN]/*K*_1-NPN_), where IC_50_ represents the concentration of the odorant required to reduce the fluorescence intensity by 50%, [1-NPN] for the free concentration of 1-NPN, and *K*_1-NPN_ for the dissociation constant of the AsOBP21f/1-NPN complex.

### In silico homology modeling and molecular docking

Molecular docking analyses were conducted to examine the interactions between AsOBP21f and its four highest-affinity ligands: decanal, pentadecanoic acid, dodecanal, and methyl tridecanoate. The protein sequence of AsOBP21f with the signal peptide removed was modeled using AlphaFold3. The chemical structures of the ligands were obtained from the NCBI PubChem database. Before docking with AutoDock Vina, the ligands and protein were preprocessed. The binding site was defined as the entire protein, with the docking center coordinates set to center_x = 4.9, center_y = −3.1, and center_z = 0.8. The cubic search space and grid spacing were set to 50 Å and 0.375 Å, respectively. Conformational sampling and scoring were conducted using a genetic algorithm, and the resulting poses were ranked on the basis of the docking scores. The top-ranked conformation was selected for binding mode analysis. After molecular docking, the protein–ligand interaction diagrams and the conformation of AsOBP21f–ligand complexes were visualized using PyMOL (DeLano Scientific, San Carlos, CA, USA). Finally, the theoretical binding free energies (Δ*G*_bind_) for the AsOBP21f and ligands were automatically calculated by the Discovery Studio client.

### EAG recordings elicited by high-affinity odorants of AsOBP21f

The odorants used for EAG recordings included decanal, pentadecanoic acid, dodecanal, and methyl tridecanoate. EAG recordings were performed as previously described [25], with the setup illustrated in Fig. S1 for clarity. Briefly, 3-day-old, non-blood-fed female mosquitoes were used for the assays; the head of each mosquito was excised under a stereomicroscope, with the distal tip of the terminal antennal segment carefully transected. The transected antenna was inserted into a glass recording electrode filled with 0.9% NaCl solution, while a reference electrode was inserted into the back of the head. The four odorant solutions were prepared in paraffin oil. A 20 μL aliquot of each odorant solution (0.001, 0.010, 0.050, 0.100, and 0.250 mg/μL for dodecanal and pentadecanoic acid; 0.01, 0.10, 1.00, 2.50, and 5.00% (v/v) for decanal and methyl tridecanoate) was applied to a 0.5 cm × 10 cm piece of filter paper, which was subsequently inserted into a 15-cm Pasteur pipette. An airflow of 10 mL/s was delivered for 0.4 s by a stimulus controller (CS-55; Syntech, the Netherlands); signals were amplified using an IDAC-2 amplifier (Syntech, the Netherlands) and recorded on a computer using Syntech EAG v2.6c software. A blank stimulation (solvent only) was used at the beginning and end of each series for baseline reference. The net EAG response for each odorant was calculated by subtracting the average amplitude of the solvent control from the average amplitude of the odorant-induced response. Each concentration was tested in triplicate, and at least six biological replicates were performed for each compound.

### Attractant effects of high-affinity odorants of AsOBP21f on mosquitoes

The attraction effect of odorants on mosquitoes was measured using a host proximity assay, based on a previously described method [8]. A schematic diagram illustrating the complete behavioral setup is provided in Fig. S2. Decanal and methyl tridecanoate were prepared at concentrations of 0.0001%, 0.001%, and 0.01% in acetone; pentadecanoic acid and dodecanal were prepared at concentrations of 0.00001 g/mL, 0.0001 g/mL, and 0.001 g/mL. Thirty 3–5-day old non-blood-fed mosquito females were starved for 12 h before being transferred to a polypropylene rearing cage (16 cm × 17.5 cm × 23 cm) for a 20-min acclimation period. An anesthetized mouse was perfumed with 1 mL test solution or acetone on the abdomen, kept at room temperature for 3 min to allow the solvent to evaporate, then placed in the middle of the gauze. The behavioral responses of mosquitoes approaching the mouse within 1 min were recorded using a camera; the response of mosquitoes to the mouse was quantified by manually counting the number of mosquitoes landing on the mouse in 1 min. The ratio of attracted mosquitoes was calculated using the following formula: percent of attracted (%) = Nl/Nt, where Nl was the number of landed mosquitoes on the mouse and Nt was the total number of mosquitoes. All the tests were repeated at least three times.

### dsRNA synthesis and RNA interference

The double-stranded RNA (dsRNA) of *AsOBP21f* was synthesized through in vitro transcription using purified PCR products containing T7 RNA polymerase promoters as templates following the manufacturer’s instructions of the T7 RiboMAX™ Express RNAi System (Promega, Madison, WI, USA). Approximately 0.5 μL of purified ds*AsOBP21f* (at a concentration of about 4 μg/μL) was injected into the abdomen of 1-day-old pupae using a Nanoject III microinjector (Drummond Scientific Company, USA), with an equivalent amount of ds*EGFP* as the control. The injected pupae were maintained at 27 ± 1 °C and 75 ± 5% relative humidity until emergence. To assess gene silencing efficiency, five adult females were collected on days 1, 2, and 3 post-emergence, respectively, and the efficiency was quantified by RT-qPCR. The primers used for dsRNA synthesis are listed in Table S1.

### Impact of AsOBP21f RNAi on host-seeking behavior

Host proximity and blood-feeding assays were performed to evaluate the impact of *AsOBP21f* RNAi on host-seeking behavior. The host proximity assay was followed the aforementioned protocol, while the blood-feeding assay was conducted according to our previously established methods [25]. Decanal and methyl tridecanoate were prepared at concentrations of 0.0001% in acetone; pentadecanoic acid and dodecanal were prepared at concentrations of 0.00001 g/mL. An anesthetized mouse was perfumed with 1 mL test solution or acetone on the abdomen, kept at room temperature for 3 min to allow the solvent to evaporate, then placed in the middle of the gauze. For each group (ds*EGFP*-injected and ds*AsOBP21f*-injected), three replicates of 30 non-blood-fed, 3-day-old female adults were starved for 12 h before being transferred to custom-made polypropylene cages (16 cm × 17.5 cm × 23 cm), which was divided into three equal compartments. An anesthetized, perfumed mouse was placed on gauze, and mosquitoes were allowed to feed for 5 min. Blood-fed and non-blood-fed mosquitoes were distinguished under microscopic examination, and the blood-feeding rate was calculated: Blood-feeding rate (%) = Nb/Nt, where Nb and Nt represent the number of blood-fed mosquitoes and the total number of mosquitoes tested, respectively. All assays were conducted between 19:00 and 21:00 at 28 °C, 75–80% relative humidity. The blood-feeding assay was repeated at least six times.

### Data analysis

Statistical analyses and graphical representations were performed using GraphPad Prism 8.0 software. Differences in *AsOBP21f* expression across tissues and the effects of various odorant doses on the attraction of wild-type or *AsOrco*^−/−^ mosquitoes were assessed using one-way analysis of variance (ANOVA). Multiple comparisons were conducted with the least significant difference (LSD) test when variances were homogeneous, or with the Tamhane test when they were heterogeneous. Differences in EAG responses between *AsOrco*^−/−^ mutants or *AsOBP21f* RNAi-treated mosquitoes and wild-type mosquitoes were analyzed using the nonparametric Mann–Whitney test, while the attractiveness of human odorants to *AsOBP21f* RNAi-treated and wild-type mosquitoes, as well as *AsOBP21f* expression levels between RNAi-treated and wild-type mosquitoes, were compared using the Student’s *t*-test. Differences were considered statistically significant at *P* < 0.05; values were presented as mean ± SD.

## Results

### Cloning and expression analysis of the AsOBP21f gene

**Fig. 1:**
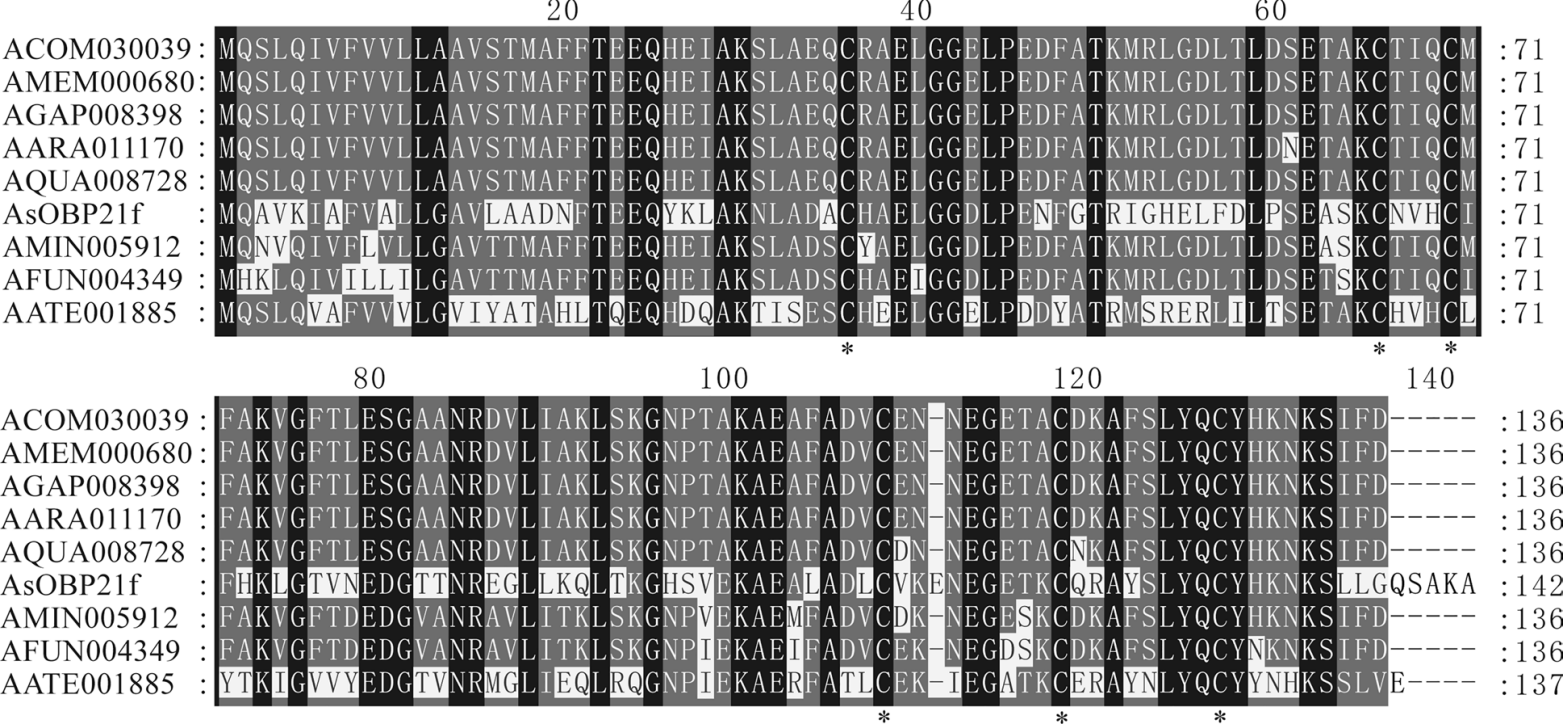
Multiple sequence alignment of amino acid sequences of AsOBP21f with OBPs from other mosquitoes. The sequences used for alignment include AsOBP21f, *Anopheles sinensis* OBP21f; ACOM030039, *Anopheles coluzzii* OBP; AMEM000680, *Anopheles merus* OBP; AGAP008398, *Anopheles gambiae* OBP21; AARA011170, *Anopheles arabiensis* OBP; AQUA008728, *Anopheles quadriannulatus* OBP; AMIN005912, *Anopheles minimus* OBP; AFUN004349, *Anopheles funestus* OBP; and AATE001885, *Anopheles atroparvus* OBP. Asterisks indicate the conserved cysteine residues

**Fig. 2:**
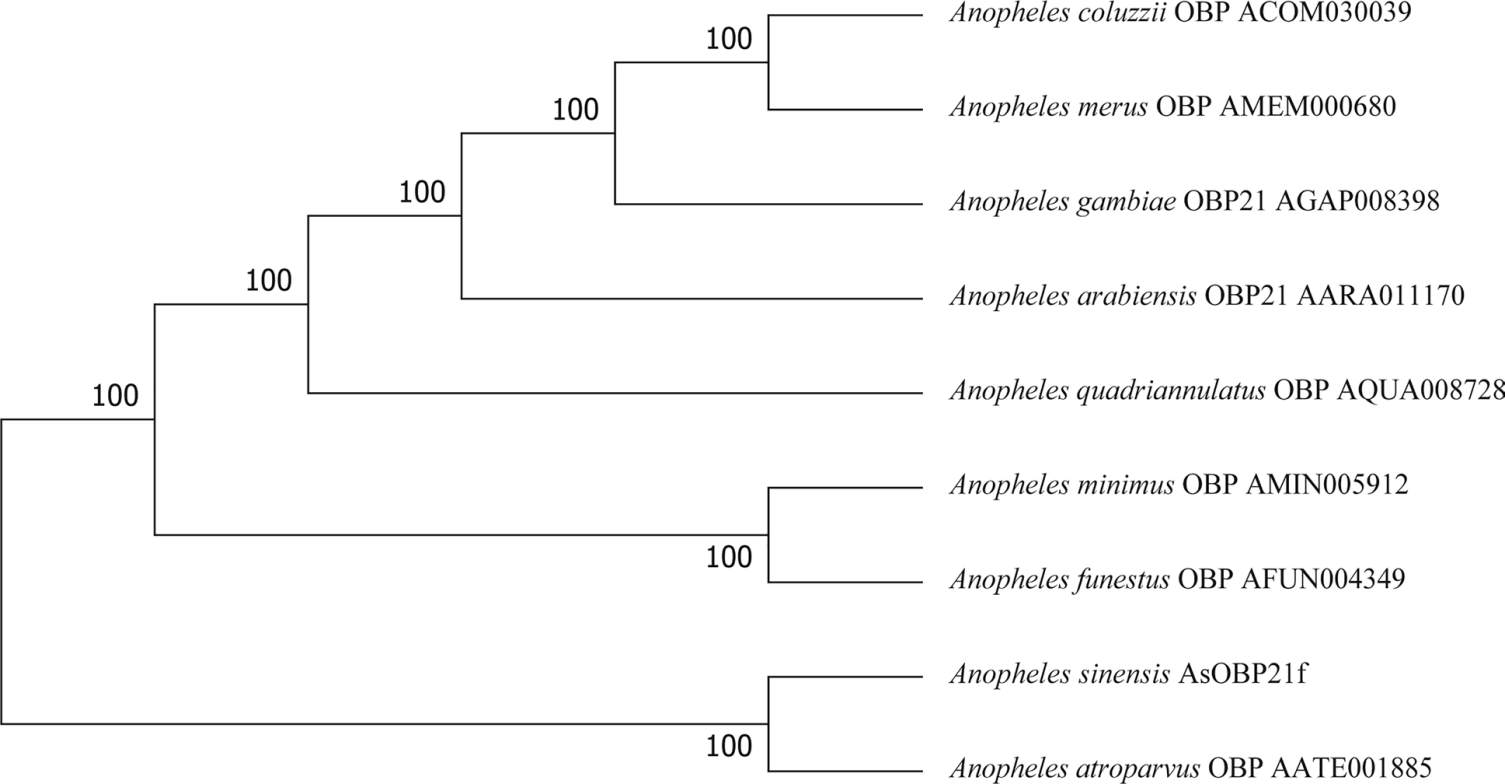
Phylogenetic tree of AsOBP21f and OBPs from other mosquitoes using the neighbor-joining method based on the amino acid sequences with 1000 bootstrappings. The OBP genes used to construct the phylogenetic tree are the same as those in Fig. 1

**Fig. 3:**
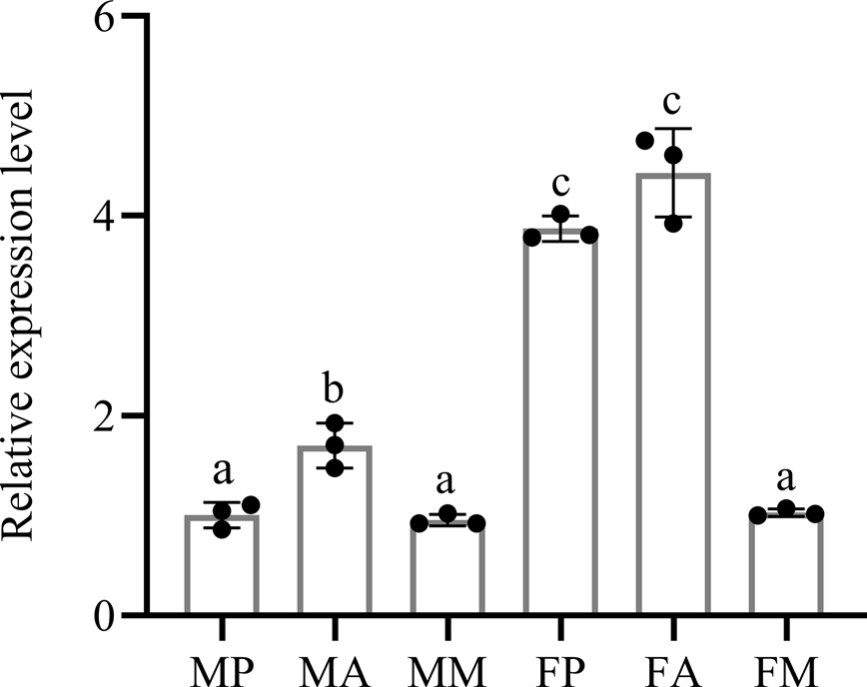
*AsOBP21f* expression levels in tissues of *Anopheles sinensis*. The relative expression levels of *AsOBP21f* were analyzed by quantitative real-time PCR and are presented as the mean ± SD of three biological replicates. FA, female antenna; FP, female proboscis; FM, female maxillary palp; MA, male antenna; MP, male proboscis; MM, male maxillary palp. The differences of *AsOBP21f* expression in different tissues were analyzed by one-way ANOVA. Different letters above the bars indicate significant differences

*AsOBP21f* is located on chromosome 2, contains no introns, and has a complete length of 495 bp encoding 142 amino acids. The predicted protein has a theoretical molecular weight of 15.48 kDa and an isoelectric point of 7.58. A 17-amino acid signal peptide was identified in the N-terminal hydrophobic region starting from the first amino acid, indicating that AsOBP21f is a secretory protein. AsOBP21f possesses the typical structural characteristics of odorant-binding proteins, including six highly conserved cysteine residues, and belongs to the Classic subfamily (Fig. 1). Phylogenetic analysis indicated that AsOBP21f grouped with several *Anopheles* OBP proteins in one clade (Fig. 2). Previous transcriptome data indicated that the *AsOBP21f* gene was predominantly expressed in olfactory-related tissues of *An. sinensis*, including antennae, proboscises, and maxillary palps [24]. Quantitative PCR analysis revealed that *AsOBP21f* expression levels in antennae were significantly higher than in other tissues. In addition, expression in female antennae and proboscises was higher than in the corresponding male tissues (Fig. 3).

### Binding analysis of AsOBP21f recombinant protein with human odorants

**Fig. 4:**
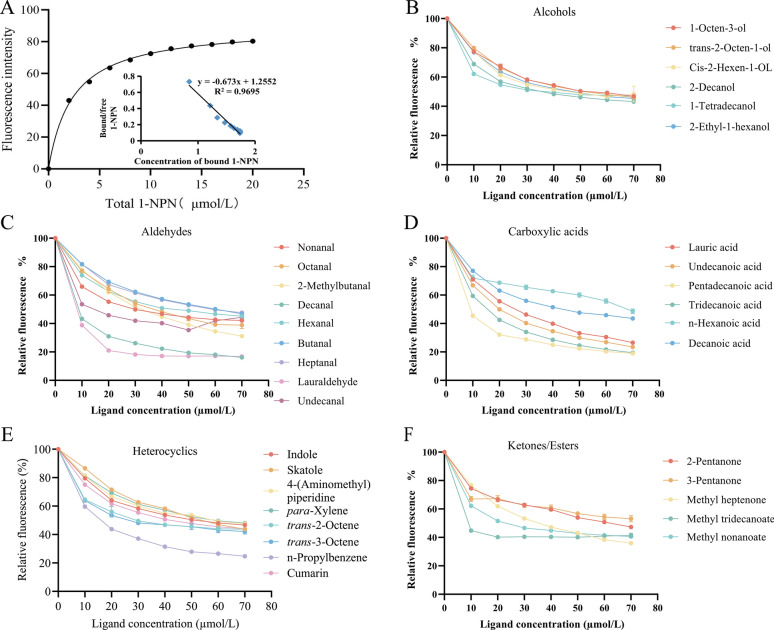
Competitive binding assays of AsOBP21f to human odorants. **A** The binding curve of AsOBP21f with 1-NPN. **B**–**F** Binding curves of *AsOBP21f* with human odorants

The recombinant AsOBP21f protein was expressed in a prokaryotic system and purified using nickel affinity chromatography for use in fluorescence competitive binding assays (Fig. S3). In these assays, 1-NPN served as a standard fluorescent reporter ligand, and the binding affinities of odor compounds to AsOBP21f were evaluated by measuring their ability to competitively displace 1-NPN from the protein–ligand complex. The results showed that the fluorescence intensity of 1-NPN bound to recombinant AsOBP21f gradually increased with rising 1-NPN concentrations, with the rate of increase slowing and eventually reaching a plateau, indicating that binding had reached saturation (Fig. 4A). Using the Scatchard equation, the dissociation constant (*K*d) between AsOBP21f and 1-NPN was determined to be 2.74 µM.

Subsequently, we further evaluated the binding capacity of *AsOBP21f* to 35 human odorants. Ten compounds reduced fluorescence intensity by more than 30% (Fig. 4B–F; Table 1), indicating that AsOBP21f can selectively bind to human odorants. Specifically, AsOBP21f exhibited strong binding affinities toward certain aldehydes, alcohols, fatty acids, and heterocyclic compounds. The highest binding affinities were observed with methyl tridecanoate, lauraldehyde, decanal, and pentadecanoic acid, with *K*i values of 2.30, 2.72, 3.98, and 4.53 µmol/L, respectively. Moderate binding capacity was observed for lauric acid, undecanoic acid, tridecanoic acid, methyl nonanoate, n-propylbenzene, and undecanal. The remaining odorants showed weak or no binding activity (Table 1).

Molecular docking confirmed that four ligands could bind to AsOBP21f, with binding free energies ranging from −2.8 to −3.7 kcal/mol. Decanal primarily binds through hydrophobic interactions with Tyr123 (Figs. S4A and S5A); dodecanal (lauraldehyde) involves hydrogen bonding with Gln127 (Figs. S4B and S5B); methyl tridecanoate similarly relies on hydrogen bonds with Ser124 (Figs. S4C and S5C); pentadecanoic acid exhibits the strongest affinity (Δ*G* =  − 3.7 kcal/mol), forming multiple hydrogen bonds primarily involving two key residues, Ser124 and Lys131 (Figs. S4D and S5D).

### EAG responses induced by four high-affinity odorants of AsOBP21f

**Fig. 5:**
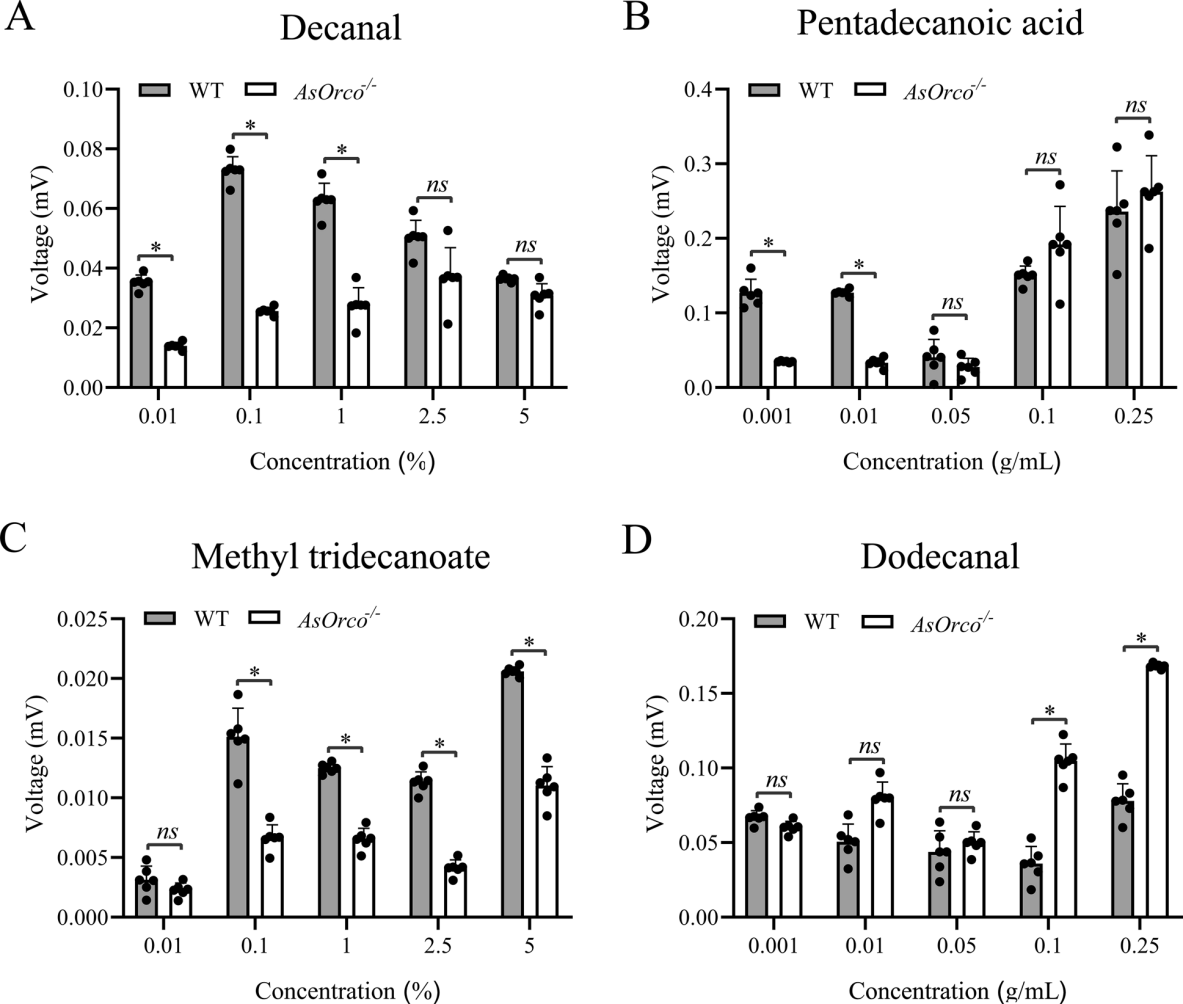
EAG response of *Anopheles sinensis* antenna to the four AsOBP21f high-affinity odorants. The EAG responses of the wild-type and *AsOrco*^−/−^ mosquitoes to different doses of decanal (**A**), pentadecanoic acid (**B**), methyl tridecanoate (**C**), and dodecanal (**D**). The Mann–Whitney test was applied for statistical analysis. Values are represented as mean ± SD (*N* = 6). ns, not significant; *P < 0.05

Next, we measured the EAG responses elicited by the four odorants that exhibited high affinity to the AsOBP21f protein. All four odorant molecules could induce EAG responses in *An. sinensis* mosquitoes, but the response intensity and dose-dependent effect among the odorants were significant differences (Fig. 5). Further comparison of EAG responses between wild-type mosquitoes and *AsOrco*^−/−^ mutants [25] revealed significant differences in their response patterns under the same stimulation conditions. Within the 0.01–1% concentration range, *AsOrco*^−/−^ mosquitoes showed significantly weaker EAG responses to decanal than wild-type mosquitoes (*Z*_0.01%_ = −2.882, *P*_0.01%_ = 0.0039; *Z*_0.10%_ = −2.887, *P*_0.10%_ = 0.0039; and *Z*_1.00%_ = −2.892, *P*_1.00%_ = 0.0038). At higher concentrations (2.5% and 5%), however, no significant differences were detected between the two groups (*Z*_2.50%_ = −1.925, *P*_2.50%_ = 0.0542; *Z*_5.00%_ = −1.283, *P*_5.00%_ = 0.1994) (Fig. 5A). Similarly, pentadecanoic acid at 0.001–0.01 g/mL evoked significantly weaker EAG responses in *AsOrco*^−/−^ mosquitoes than in wild-type mosquitoes (*Z*_0.001 g/mL_ = −1.964, *P*_0.001 g/mL_ = 0.0495; *Z*_0.010 g/mL_ = −2.121, *P*_0.010 g/mL_ = 0.0339) (Fig. 5B). Methyl tridecanoate at 0.1–5% also elicited markedly reduced EAG responses in *AsOrco*^−/−^ mosquitoes compared with wild-type controls (*Z*_0.10%_ = −2.121, *P*_0.10%_ = 0.0339; *Z*_1.00%_ = −1.964, *P*_1.00%_ = 0.0495; *Z*_2.50%_ = −1.964, *P*_2.50%_ = 0.0495; and *Z*_5.00%_ = −1.121, *P*_5.00%_ = 0.0339) (Fig. 5C). In contrast, dodecanal induced significantly stronger EAG responses in *AsOrco*^−/−^ mosquitoes than in wild-type mosquitoes at concentrations of 0.1–0.25 g/mL (*Z*_0.100 g/mL_ = −2.121, *P*_0.100 g/mL_ = 0.0339; *Z*_0.250 g/mL_ = −1.964, *P*_0.250 g/mL_ = 0.0495) (Fig. 5D).

### Attraction effects of four AsOBP21f high-affinity odorants to *An. sinensis*

**Fig. 6:**
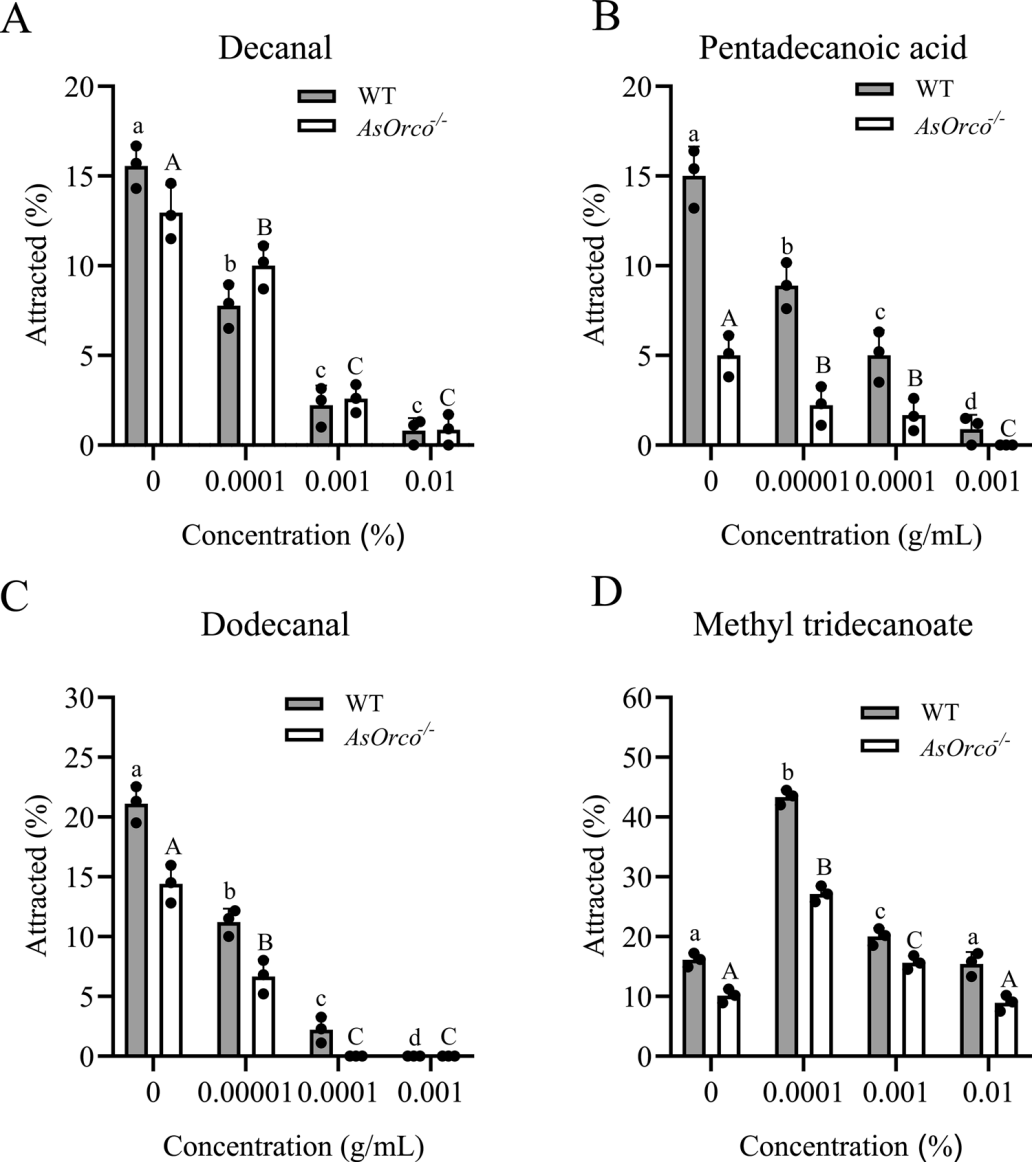
The attraction of human odorants to *Anopheles sinensis.* The attraction effects of decanal (**A**), pentadecanoic acid (**B**), dodecanal (**C**), and methyl tridecanoate (**D**) on wild-type and *AsOrco*^−/−^ mosquitoes were measured using a host proximity assay. The effects of different odorant doses on the attraction of wild-type and *AsOrco*^−/−^ mosquitoes were analyzed by one-way ANOVA, and comparisons between genotypes were made using Student’s *t*-test. Different uppercase letters above the bars indicate significant differences in the attraction of *AsOrco*^−/−^ mutants to human odorants at different doses, whereas different lowercase letters above the bars indicate significant differences in the attraction of wild-type mosquitoes to human odorants at different doses

The attraction effects of the four odorants on *An. sinensis* were assessed using host proximity assays. Methyl tridecanoate, dodecanal, decanal, and pentadecanoic acid exhibited significant regulatory effects on the behavior of *An. sinensis* (Fig. 6). Decanal showed strong repellent effects in both wild-type and AsOrco^−/−^ mosquitoes (wild-type: *F* = 139.275, *P* < 0.00001; *AsOrco*^−/−^: *F* = 77.470, *P* < 0.00001) (Fig. 6A). Dodecanal (wild-type: *F* = 235.678, *P* < 0.00001; *AsOrco*^−/−^: *F* = 15.595, *P* = 0.0011) and pentadecanoic acid (wild-type: *F* = 62.214, *P* < 0.00001; *AsOrco*^−/−^: *F* = 27.021, *P* = 0.0002) exhibited similarly pronounced repellent effects (Fig. 6B, C). Notably, methyl tridecanoate demonstrated a significant attractant effect at low concentrations (wild-type: *F* = 238.505, *P* < 0.00001; *AsOrco*^−/−^: *F* = 123.583, *P* < 0.00001) (Fig. 6D).

### Effect of AsOBP21f RNAi on mosquito host-seeking behavior

**Fig. 7:**
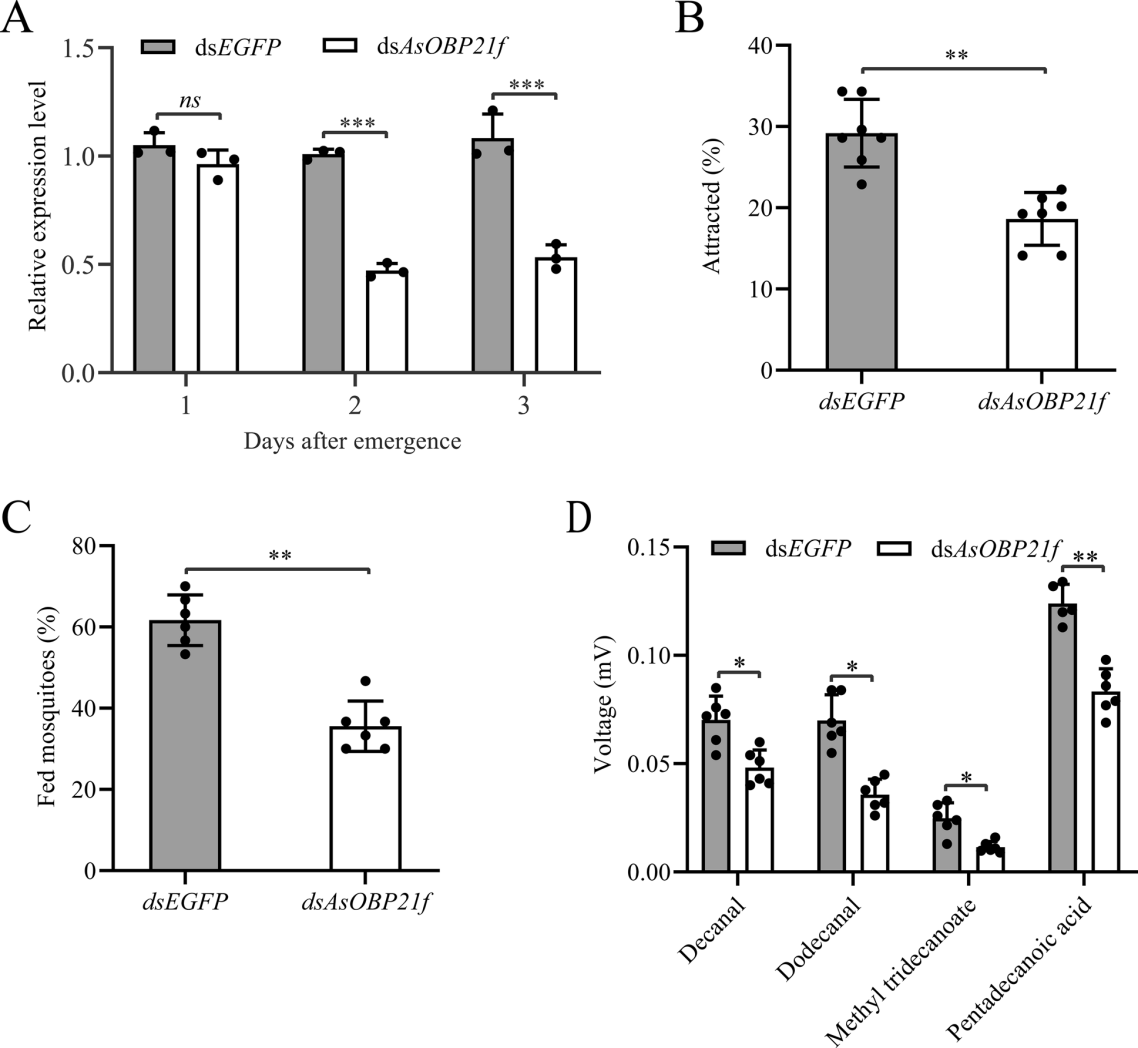
The effect of *AsOBP21f* gene RNAi on host-seeking behavior in *Anopheles sinensis*. **A** Effect of RNAi on the expression levels of *AsOBP21f*. **B** A percentage of mosquitoes attracted to a human arm in 1 min was measured by the human host proximity assay. **C** The blood-feeding rate of control and RNAi-treated mosquitoes after feeding on mice for 5 min (six biological replicates for each group, with 30 mosquitoes per replicate). **D** The antennal electrophysiological responses of identified compounds in *An. sinensis*. EAG amplitudes were calculated by dividing the amplitudes of blank controls (*N* = 6). Asterisks indicate significant differences between the ds*AsOBP21f* and ds*EGFP*-treated groups. *P < 0.05, **P < 0.01, ***P < 0.001

We conducted functional validation using RNAi to further investigate the role of *AsOBP21f* in the host-seeking behavior of *An. sinensis*. The results showed that, compared with the control group injected with ds*EGFP*, the expression levels of *AsOBP21f* in RNAi-treated females decreased by 6.3%, 52.5%, and 46.7% on days 1, 2, and 3 post-emergence, respectively (Fig. 7A). Subsequently, host proximity assays were performed to assess the host-seeking behavior of mosquitoes on day 2 post-emergence following injection with ds*AsOBP21f* or ds*EGFP*. The proportion of *AsOBP21f* RNAi-treated females approaching a mouse was significantly lower than that of the control mosquitoes (*Z* = −3.141, *P* = 0.0017) (Fig. 7B). The blood-feeding rate of *AsOBP21f* RNAi-treated mosquitoes within 5 min was significantly lower than that of the control mosquitoes injected with ds*EGFP* (*Z* = −2.892, *P* = 0.0038) (Fig. 7C).

Furthermore, EAG recordings revealed that the electrophysiological responses of RNAi-treated females to decanal (*Z* = −2.647, *P* = 0.0081), dodecanal (*Z* = −2.887, *P* = 0.0039), methyl tridecanoate (*Z* = −2.647, *P* = 0.0081), and pentadecanoic acid (*Z* = −2.739, *P* = 0.0062) were significantly reduced (Fig. 7D).

## Discussion

Mosquitoes primarily rely on olfactory cues to detect human-derived odorants for host seeking [3, 27]. Their preference for certain individuals within human populations is associated with differences in the composition of body odor components [28, 29]. Therefore, identifying human odorants that attract mosquitoes, deciphering the olfactory genes responsible for detecting these compounds, and elucidating the activated neural signaling pathways will help uncover the chemical ecology and molecular mechanisms underlying mosquito host-seeking behavior [27]. This study demonstrated that *AsOBP21f* is involved in the host-seeking behavior of *An. sinensis* by mediating the detection of human odorants such as methyl tridecanoate, thereby providing a potential target for malaria control.

The clade formed by *AsOBP21f* with homologous OBP21 proteins from other *Anopheles* species suggests its conserved role in the olfactory systems of *Anopheles* mosquitoes (Fig. 2). This protein exhibits characteristics of the Classic OBP subfamily: three pairs of disulfide bonds formed by six conserved cysteine residues and a hydrophobic binding pocket composed of α-helices (Fig. 1; Fig. S4). In addition, *AsOBP21f* expression was significantly higher in female antennae than in males (Fig. 3); a similar pattern was observed in other mosquito species. For example, of the 55 OBP genes in *An. gambiae*, 29 were expressed in antennae, with 8 having female-specific expression [30]. In *Culex quinquefasciatus*, *CquiOBP1* and *CquiOBP2* exhibited antenna-specific expression, while other OBP genes showed non-olfactory organ or broad expression [31, 32]. This sexual dimorphism of OBP gene expression aligns with their roles in female-specific behaviors, such as host-seeking and oviposition site selection [8, 33–35]. As expected, RNAi-mediated knock-down of *AsOBP21f* expression resulted in significantly weaker EAG responses of the mosquito antenna to human odorants, reduced host approach rates (Fig. 7B), and dampened blood-feeding frequency compared with wild-type mosquitoes (Fig. 7C). These results demonstrate that *AsOBP21f* is involved in blood feeding via mediating host-seeking behavior in *An. sinensis* mosquitoes.

To further elucidate the chemosensory mechanism by which AsOBP21f contributes to host-seeking behavior, we analyzed its interactions with a range of human odor molecules. The results indicated that AsOBP21f exhibits a selective binding preference for certain human-derived odorants, showing particularly strong affinities for methyl tridecanoate, dodecanal, decanal, and pentadecanoic acid (Table 1). These revealed that AsOBP21f exhibited a binding preference for hydrophobic molecules containing C10–C15 carbon chains, particularly those containing aldehyde, ester, or carboxyl functional groups, demonstrating the selective recognition capabilities for such compounds. It should be noted, however, that only 35 compounds were tested in this study; AsOBP21f may recognize a broader range of ligands or bind other molecules with even higher affinities.

Molecular docking further demonstrated that AsOBP21f engages various ligands through specific amino acid residues (Fig. S5). This structural adaptability enables the protein to accommodate hydrophobic molecules with carbon chain lengths ranging from C10 to C15, offering new insight into the diversity of ligand recognition within the OBP family. Notably, among the compounds that showed high affinity for AsOBP21f, decanal (Fig. 6A), pentadecanoic acid (Fig. 6B), and dodecanal (Fig. 6C), exhibited repellent activity, whereas methyl tridecanoate acted as an attractant (Fig. 6D). The influence of these dose-dependent effects on mosquito host-seeking behavior warrants further investigation. Interestingly, high concentrations of dodecanal elicited stronger responses in *AsOrco*^−/−^ mutants than in wild-type mosquitoes, which suggests the involvement of a noncanonical olfactory perception pathway (Fig. 5D). This may be due to changes in the expression of ion channel signaling-related genes caused by the *AsOrco* gene mutation [36], or it may result from a sensory compensation mechanism [37]; the exact cause requires further investigation.

Our findings suggest that *An. sinensis* employs a combinatorial OBP-based recognition system to detect host-associated odors. For instance, indole is recognized through the combined actions of AsOBP1/2/62, whereas decanal, heptanal, and dodecanal are detected by AsOBP2/21f, AsOBP1/2, and AsOBP1/21f, respectively [8, 38, 39]. Such a redundant recognition network likely enhances the reliability of odor detection in complex and variable environments. By leveraging specific combinations of OBPs, mosquitoes not only improve the precision of host identification but also accommodate individual differences in odor profiles. This hypothesis offers a molecular framework for understanding the mechanisms underlying mosquito host detection.

Among the redundant OBP ligands in *An. sinensis*, indole is not only a volatile compound found in mosquito oviposition sites but also a primary component of human volatiles, accounting for nearly 30% of the total headspace volatiles in human sweat [6]. It can strongly activate the AgOR2 olfactory receptor in *An. gambiae* [40]. Decanal and heptanal are common aldehydes found in the volatile emissions from the skin of animals such as sheep, cattle, donkeys, goats, and humans [41]. These compounds can elicit antennal EAG responses in mosquitoes and exhibit attractive effects [42, 43]. Dodecanal, a major component of human foot odor, also triggers EAG responses in *An. gambiae* and demonstrates attractive activity for this species [43]. These results highlight the critical roles of indole, decanal, heptanal, and dodecanal in mosquito host-seeking behavior.

## Conclusions

This study demonstrates that *AsOBP21f* plays a key role in the host-seeking behavior of *An. sinensis*. Given the essential role of blood-feeding behavior for *An. sinensis* and other mosquito vectors to acquire and transmit pathogens, the function of *AsOBP21f* in host-seeking makes it an ideal target candidate for malaria control and potential intervention strategies can be developed on the basis of this gene.

## Supplementary Information

**Additional file 1.**

## Data Availability

Data supporting the main conclusions of this study are included in the manuscript.
