# Supplementary material for: Functional characterization of AsOBP21f, an odorant-binding protein involved in human odor detection in the malaria vector Anopheles sinensis
Source: Parasit Vectors. 2026 Mar 23;19:196. doi: 10.1186/s13071-026-07333-0 (PMC13134246; doi:10.1186/s13071-026-07333-0)
Supplement: Supplementary file 1 — Additional file 1. [file 13071_2026_7333_MOESM1_ESM.docx]

Table S1 Primers used in this study

| Primer name | Primer sequence (5’-3’) | Usage of primers |
| --- | --- | --- |
| qOBP21f-F | GCCCAGTGAGGCTTCTAAGG | qPCR primers |
| qOBP21f-R | GTTCCGTCCTCGTTTACGGT |  |
| RpS7-F | CGGAGAAGATGGCATGGGAGAT | Reference gene primers |
| RpS7-R | ATAGTGAGCATAGGCCCGGTTA |  |
| OBP21f-F | GCGGGGAAACAAACATCAGG | Gene cloning primers  Prokaryotic expression vector construction primers  OBP21f RNAi primers  EGFP RNAi primers |
| OBP21f-R  OBP21f-pF  OBP21f-pR  OBP21f-IF  OBP21f-IR  EGFP-IF  EGFP-IR | CGCTGATTGCTCATGAATCGA  GCACCATGGGTGAGTAGAAAATACAAAAC  ATCCTCGAGAAGCCTTCGCGGACTGGCCA  TAATACGACTCACTATAGGGAGTGTAAAG ATCGCTTTCGTCGC  TAATACGACTCACTATAGGGAGGGCACTT CGTTTCGCCTTC  TAATACGACTCACTATAGGGAGCGTAAACGGCCACAAGTTCAG  TAATACGACTCACTATAGGGAGCTTCTGCTTGTCGGCCATGA |  |

The underlined sequences indicate the restriction endonuclease sites.

**
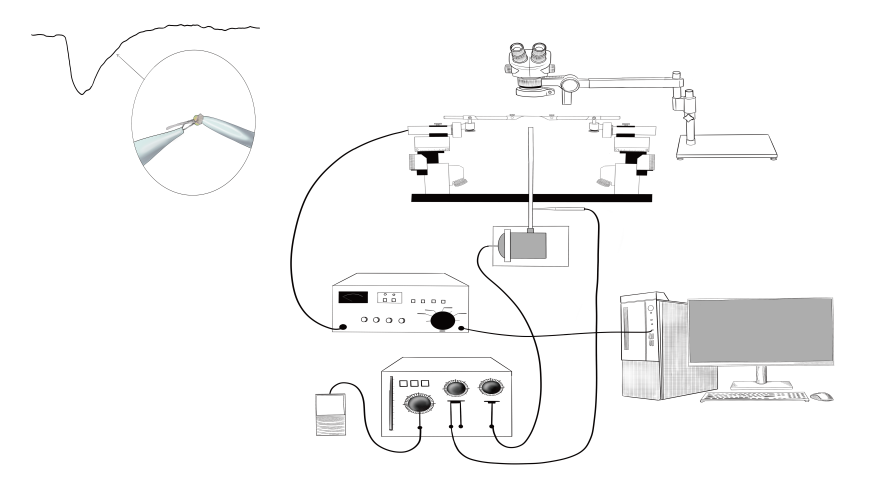
**

**Fig S1. Schematic diagram of the Electroantennography (EAG) recording setup.**

**

**

**Fig S2. Schematic of the host proximity assay for mosquito behavioral analysis.**

**
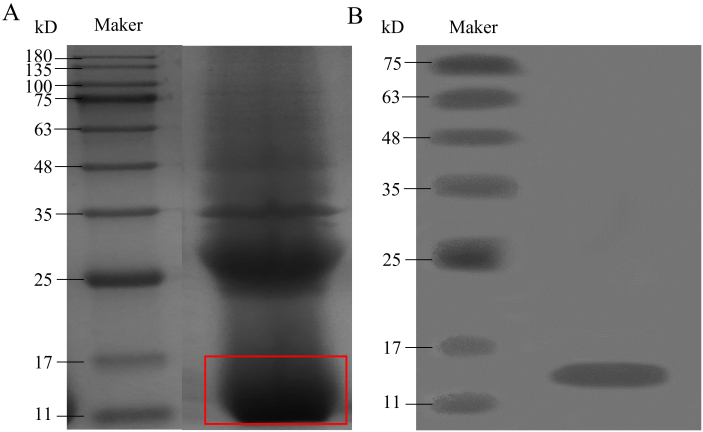
**

**Figure S3** SDS-PAGE analysis of expression (A) and purification (B) of AsOBP21f

**
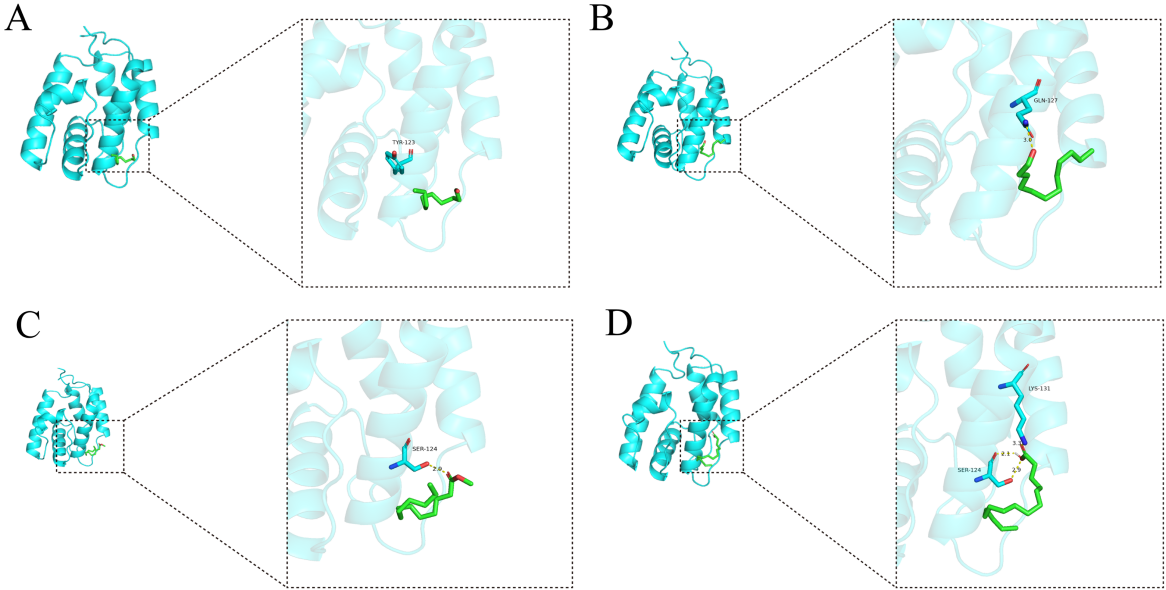
**

**Figure S4** The molecular docking results of *AsOBP21f* with decanal (A), dodecanal (B), methyl tridecanoate (C), pentadecanoic acid (D).


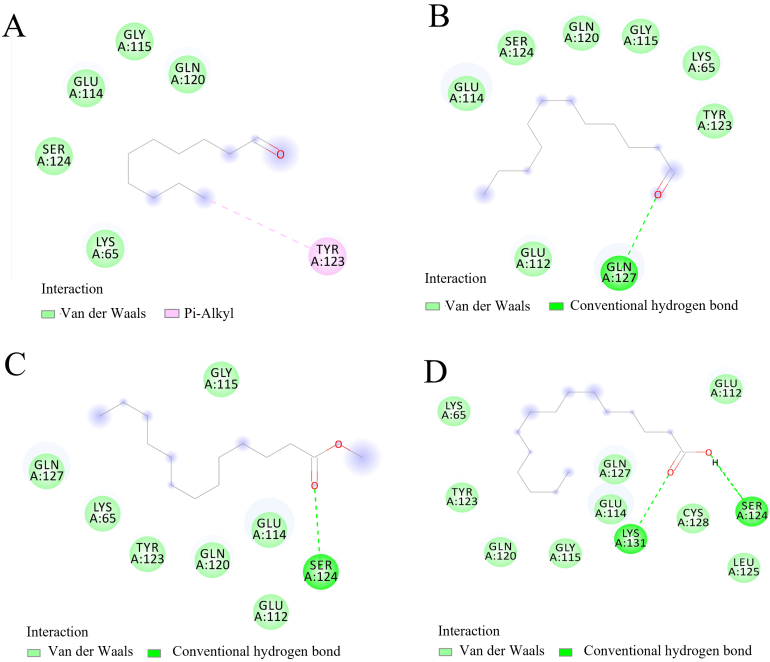


**Figure** **S5** Interaction diagram of amino acid residues to ligands. (A) Decanal, (B) dodecanal, (C) Methyl tridecanoate, (D) Pentadecanoic acid.
